# Investigating B Cell Development, Natural and Primary Antibody Responses in Ly-6A/Sca-1 Deficient Mice

**DOI:** 10.1371/journal.pone.0157271

**Published:** 2016-06-20

**Authors:** Morgan A. Jones, Sean DeWolf, Vimvara Vacharathit, Michelle Yim, Stacey Spencer, Anil K. Bamezai

**Affiliations:** Department of Biology, Villanova University, 800 E. Lancaster Avenue, Villanova, PA, 19085, United States of America; McGill University, CANADA

## Abstract

Ly-6A/Stem cell antigen-1 (Ly-6A/Sca-1) is a glycosylphosphatidylinositol-anchored protein expressed on many cell types including hematopoietic stem cells (HSCs) and early lymphoid-specific progenitors. Ly-6A/Sca-1 is expressed on CD4^+^ T cells and plays a role in regulating cellular responses to foreign antigens. The role of Ly-6A/Sca-1 in primary antibody responses has not been defined. To investigate whether Ly-6A/Sca-1 functions in humoral immunity, we first injected Ly-6A/Sca-1-deficient and wild-type control mice with chicken ovalbumin (c-Ova) protein mixed with an adjuvant. We then assessed the ability of the mice to generate a primary antibody response against cOva. We further examined the development of B cells and circulating antibody isotypes in non-immunized Ly-6A/Sca-1deficient mice to determine if Ly6A/Sca-1 functions in development irrespective of antigen-specific immune activation. Ly-6A/Sca-1/Sca-1-deficient mice did not show any significant changes in the number of B lymphocytes in the bone marrow and peripheral lymphoid tissues. Interestingly, Ly-6A/Sca-1/Sca-1^-/-^ mice have significantly elevated serum levels of IgA with λ light chains compared to wild type controls. B cell clusters with high reactivity to anti-IgA λ monoclonal antibody were detected in the lamina propria of the gut, though this was not observed in the bone marrow and peripheral lymphoid tissues. Despite these differences, the Ly-6A/Sca-1deficient mice generated a similar primary antibody response when compared to the wild-type mice. In summary, we conclude that the primary antibody response to cOva antigen is similar in Ly-6A/Sca-1deficient and sufficient mice. In addition, we report significantly higher expression of the immunoglobulin λ light chain by B cells in lamina propria of Ly-6A/Sca-1deficient mice when compared to the wild-type control.

## Introduction

Ly-6A/Sca-1 (also known as Stem cell antigen-1 and T cell Activating Protein) is a GPI (Glycosyl-phosphatidylinositol) anchored protein that belongs to a Ly-6 supergene family [1, 2]. Ly-6 proteins are expressed both in invertebrates and vertebrates [3–6]. Mouse Ly-6 proteins are markers of differentiation of immune cells and have cell adhesion and cell signaling properties [7–12]. Ly-6A/Sca-1, is expressed on primary CD4^+^ T cells at low levels and is up-regulated during activation [13–15]. Experiments examining antigen receptor responses of CD4^+^ T cells lacking expression of Ly-6A/Sca-1 or precociously expressing higher level of this protein indicate its inhibitory role in immune activation [11, 12]. CD4^+^ T cells lacking the expression of Ly-6A/Sca-1 on the surface show moderately high responsiveness to signaling through the TCR [11] and overexpression of this protein on primary CD4^+^ T cells results in their reduced responsiveness in response to a model antigen [12]. In addition, primary CD4^+^ T cells with altered expression of Ly-6A/Sca-1 show a distinct cytokine profile [12] but its role in aiding primary antibody response is unknown. Effector helper T cells play a central role in T-dependent B cell responses that require cognate interaction between effector T cells with antigen-primed B cells [16]. B cells present the antigen to effector helper T cells [16] and a number of co-stimulatory proteins on B cells interact with their cognate ligands on effector T cells leading up to a germinal center reaction and development of generation of memory B cells and plasma cells [17]. Constitutive expression of Ly-6A/Sca-1 on 35% of primary CD4^+^ T cells and upregulated expression upon activation provides a strong rationale to study primary antibody responses in Ly-6A/Sca-1 deficient mice.

High surface expression of Ly-6A/Sca-1 is observed on the earliest hematopoietic progenitors, the hematopoietic stem cells (HSC), which possess self-renewal capacity and the potential to differentiate into every blood lineage [18]. Bone marrow multipotent progenitors (MPPs), which can differentiate into the majority of blood lineages but lack self-renewal potential, also express high levels of Ly-6A/Sca-1 protein in conjunction with c-Kit and flt3 proteins [19–20]. The common lymphoid progenitors (CLPs), which develop from the MPPs and have a lin^neg^Sca1(Ly-6A)^low^kit^low^flt3^pos^IL7Ra^pos^ phenotype, are predominantly committed to generating lymphocytes [21]. A body of data suggests that the CLPs are capable of developing into B [22–24], T [22, 24, 25], NK [26] and dendritic cells [23, 27]. While there is an agreement on CLPs for B cell development [28, 29], a controversy still exists about their role as precursors to T cell development [24, 25]. Other investigators have identified lin^neg^Sca1(Ly-6A)^low^kit^neg^IL7Ra^pos^Flt3^pos^ precursor cells that are distinct from classical CLPs but possess T, B, and NK cell potential [30, 31]. More recently Ly-6D^+^ expressing CLPs exclusively generate B cells [32]. An understanding of the functional role of Ly-6A/Sca-1 in influencing the differentiation of early progenitors, especially in the development of B lymphocytes, is limited. Using the Ly-6A/Sca-1deficient mouse model, it was observed that Ly-6A/Sca-1 expression plays a role in efficient production of megakaryocytes and platelets in the bone marrow [33]. Our studies using the Ly-6A/Sca-1 deficient mouse model have shown that regulated expression of Ly-6A/Sca-1on developing T cells is important for T cell development in mice [34]. More importantly, the status of B cell development in Ly-6A/Sca-1^-/-^ mice is unknown. These experiments were critical for assessing and interpreting our findings that relate to antigen-specific primary antibody responses in Ly-6A/Sca-1deficient mice. We find that B cell development in Ly-6A/Sca-1 deficient mice is not altered. Interestingly, we observed a higher representation of IgA with λ light chain in the blood as well as λ^+^ B cells in the lamina propria of Ly-6A/Sca-1 deficient mice. Regardless of these changes, the primary antibody response to chicken ovalbumin mounted by Ly-6A/Sca-1 deficient and sufficient mice was similar.

## Materials and Methods

### Mice

Ly-6A/Sca-1 knockout mice (a generous gift from Dr. Patrick Flood at University of North Carolina, Chapel Hill) [11] bred on a C57BL/6 background and C57BL/6 wild-type mice (Taconic, Germantown, NY, USA) of both sexes (ages 5–8 weeks) were used for the experiments. Mice were housed and bred at the Villanova University vivarium in accordance with approved IACUC protocols and guidelines. The use of animals was approved by The Villanova Institutional Animal Care committee (IACUC) under protocol “BP05.BIO”.

### Media and buffers

Wash media used in this research was a RPMI 1640 medium (Invitrogen, Grand Island, NY, USA) containing 5% heat-inactivated fetal bovine serum (Atlanta Biologicals, Norcross, GA, USA), 2 mM HEPES (Invitrogen, Grand Island, NY, USA), and 1% antibiotic-antimycotic (Invitrogen, Grand Island, NY, USA). Other reagents used were: paraformaldehyde (Sigma Aldrich, St. Louis, MO, USA), Tris-NH4Cl (Sigma Aldrich, St. Louis, MO, USA) and Phosphate Buffered Saline (PBS), pH 7.2–7.4 (Fisher Scientific, Pittsburgh, PA, USA and EM Science, Gibbstown, NJ, USA).

### Preparation of cells from thymus, bone marrow, spleen, and peritoneum

Thymus, spleen and lymph nodes removed from the mice were deposited into 5 mL of cold wash media and gently ground using frosted ends of glass slides. Bone marrow cells (from the tibia, fibula, femur and humerus) was extracted by repeatedly flushing with 3 mL of cold wash media using a 25 gauge needle attached to a 3 cc syringe (BD Biosciences, East Rutherford, NJ, USA). Samples were centrifuged for 10 minutes at 1000 rpm at 4°C and the supernatant was aspirated. Red blood cells in these cell preparations from spleen and bone marrow were subjected to lysis by incubating the cells in 1.0 mL of hypotonic Tris-NH_4_Cl solution at 37°C for 5 minutes. For harvesting cells from the peritoneal exudates, 10 ml of cold serum-free RPMI 1640 was injected into the peritoneum using 18 gauge needle. The injected medium was withdrawn and deposited into a 15 mL tube on ice and harvested after centrifugation for 10 minutes at 1000 rpm at 4°C. To isolate peyer’s patches (PP), female and male Ly6A/Sca-1^-/-^ and C57Bl/6 wild type (control) mice were euthanized with CO_2_ asphyxiation. The small intestine was dissected by cutting intestine ∼0.5 cm below stomach and then holding the intestine at the top with forceps and slowly pulling it out of the peritoneal cavity and incising it ∼1 cm above cecum. The intestines were pulled gently from the peritoneal cavity, separating unwanted fat and connective tissue. Contents of the intestine were removed by squeezing with 2 pairs of tweezers, gently along the length to expel the contents. Peyer’s patches (PPs) were visualized using feces as background. The PPs were removed with fine forceps and scissors by grasping the patches in the tips of the forceps and cutting them off with scissors. Peyer’s patches resemble a tiny, single “node”, two to eight PPs were found in a small intestine.

### Immunizations and serum collection

Chicken ovalbumin was dissolved in phosphate-buffered saline (PBS) at 2 mg/mL concentration and filtered with 0.45μm membrane filter and mixed 1:1 with TiterMax Gold (TiterMax USA, Atlanta, USA). About 4 week old male Ly-6A/Sca-1^-/-^ or wild-type mice were injected with 100 μL OVA and TiterMax, 100 μL TiterMax alone intraperitoneally. Animals were housed in the animal facility for 21 days post-injection and then euthanized by CO_2_ inhalation. Blood was collected via cardiac prick and allowed to coagulate at 4°C. Serum was collected after centrifugation at 1000 rpm for 10 minutes at 4°C. Control normal serum was obtained from un-injected, age and sex-matched mice.

### Flow cytometric analyses

After the cells had been prepared, between 500,000 and 1.25 million cells from these single-cell suspensions were incubated with 1 μg of the appropriate primary antibodies (listed below) for 45 minutes on ice, with vortexing at every 7–10 minute intervals. These cells were washed twice with phosphate buffer followed by incubation for 15 minutes with an appropriate second step reagent-conjugated to a fluorochrome. After 15 minutes the cells were washed twice with PBS, and re-suspended in 1XPBS and fixed with paraformaldehyde at 1% final concentration. 10,000 and 25,000 cells were analyzed with a Becton Dickinson FACS Calibur (BD Biosciences, San Jose, CA, USA). Primary antibodies used in this investigation were: Anti-CD3-FITC, anti-CD3-PE anti-CD4-FITC, anti-CD4-PE, anti-CD44 Biotin, anti-B220-FITC and anti-IgM-PE (BioLegend Inc., San Diego, CA, USA). Also anti-CD25-PE, anti-CD5-PE, anti-IgM-PE, anti-IgA-FITC, and anti-B220-PerCP (BD Biosciences, San Jose, CA, USA). Secondary antibodies used were: Streptavidin-PerCP (BioLegend Inc, San Diego, CA, USA) and Streptavidin-PE (BD Biosciences, San Jose, CA, USA).

### Serum ELISAs

96 well plates were coated with 100 μL of 10 μg/mL OVA in carbonate-bicarbonate buffer (pH9.6) by incubating for overnight at 4°C. Next day the plates were washed 3 times with Tris-buffered saline (TBS) containing 0.1% Tween 20 and non-specific siteslates were blocked 1% BSA in TBS buffer for overnight at 4°C. The wells were washed thrice with TBS with Tween-20 before incubation with diluted serum from OVA immunized mice. 100 μL of serum from each of the immunized animals or normal serum was added in duplicate to the plate at 1:30, 1:90, 01:270, and 1:810 serial dilutions in TBS with 1% BSA. Samples were incubated for 1 hour at 37°C. Assay wells were washed 5 times with TBS-Tween and incubated with 100 μL either alkaline phosphatase conjugated anti-IgM (1:1000), anti-IgG1 (1:1000), anti-IgG2a (1:3000), or anti-IgA (1:12000) (Sigma- Aldrich, St-Louis, MO) detection reagent. Assay wells were incubated for 1 hour at 37°C and washed 5 times as described previously. An alkaline phosphatase amplification kit was then used according to the manufacturer’s recommendations (Life Technologies, Grand Island, NY) to develop the ELISAs and samples were analyzed with the plate reader SpectraMax 190 (Molecular Devices, Sunnyvale, CA).

### Antibody isotype analyses by cytometric bead array (CBA)

Sera from unimmunized Ly-6A/Sca-1^-/-^ and wild-type female C57bl/6 mice, age 5–6 weeks were diluted 1:10 in CBA Master Buffer (BD assay kit) and 50 μL was added to the CBA as per protocol (BD Biosciences, East Rutherford, NJ). This allows the binding of fluorescent beads specific to individual heavy (μ, γ1, γ2a, γ2b, γ3, α, ε) and light chains (κ and λ) immunoglobulins present in the serum. A total of 7 mice were analyzed per genotype. Samples were analyzed by flow cytometry allowing for the identification of these different serum isotypes and light chain usage.

### Tissue sectioning and immunohistochemistry

Ileum from female Ly6A/Sca-1^-/-^ and female wild-type C57Bl/6 mice (4 per genotype) of 4–8 weeks of age was dissected and the tissue were embedded in Optimal Cutting Compound (OCT) (Sakura, Torrance, CA), and frozen over dry ice and stored at -80°C until their sectioning. A Leica cryostat (GMI, Ramsey, MN), at -24°C, was used to cut and mount frozen serial sections, 7 μm thick, on 3-welled glass slides of size 22x50mm (Electron Microscopy Sciences, Hatfield, PA). The slides were allowed to air-dry, fixed in 60%, 70%, 80%, and 90% acetone (EMD Chemicals Inc, Gibbstown, NJ) for 3 minutes each consecutively, air-dried overnight, and stored at -80°C. For immunostaining with antibodies, the tissue sections were rehydrated in Tris-Phosphate Buffered Saline (TPBS) mixed with 0.005% Tween in a humidifying chamber, followed by incubation with an avidin/biotin blocking kit (Vector Laboratories, Burlingame, CA) to block endogenous biotin. The kit required 15 minutes of incubation by avidin followed by 15 minutes of incubation by biotin. Each well was incubated with 25μg/ml of biotinylated primary antibody for one hour to localize specific proteins in the cells of the tissue sections. For detection of IgA, expressing both κ and λ light chain or only λ light chain, the sections were incubated with primary biotinylated anti-IgA and anti-Igλ (BioLegend, Sand Diego, CA). For characterization of the wild-type and Ly-6 knockout mice, anti-Ly-6A/Sca-1, anti-CD119 (receptor for interferon γ—control), and anti-Thy1.2 were used (BD Biosciences,San Jose, CA; BioLegend, Sand Diego, CA). Following 3 rinses in TPBS for 5 minutes each, sections were incubated with 0.3% H_2_O_2_ for 30 minutes. Sections were rinsed 3 times again with TPBS and developed in Vectastain Elite ABC reagent (Vector Laboratories, Burlingame, CA 94010) for 30 min. Following 3 rinses in TPBS, staining was visualized by adding chromogen 3’- diaminobenzidine (DAB, Vector Laboratories, Burlingame, CA 94010) for 3 minutes. The sections were then counterstained with Hematoxylin QS (Vector Laboratories, Burlingame, CA 94010) for 20 seconds to see cellular features, mounted with Paramount, and visualized using a basic bright field light microscope fitted with an Olympus digital camera. Images were captured by Spot Advancement software at 40x and 100x magnification.

### Statistics

A two way ANOVA between sex and genotype was performed for each tissue being analyzed as well as each stage of development being analyzed for B and T lymphocytes. Post hoc Tukey’s HSD tests were performed on ANOVA to determine the significance of the results. Significant interactions between the genotype, age and sex were determined and reported here.

## Results

### Unaltered development of B and T cells in Ly-6A/Sca-1 deficient mice

**Fig 1:**
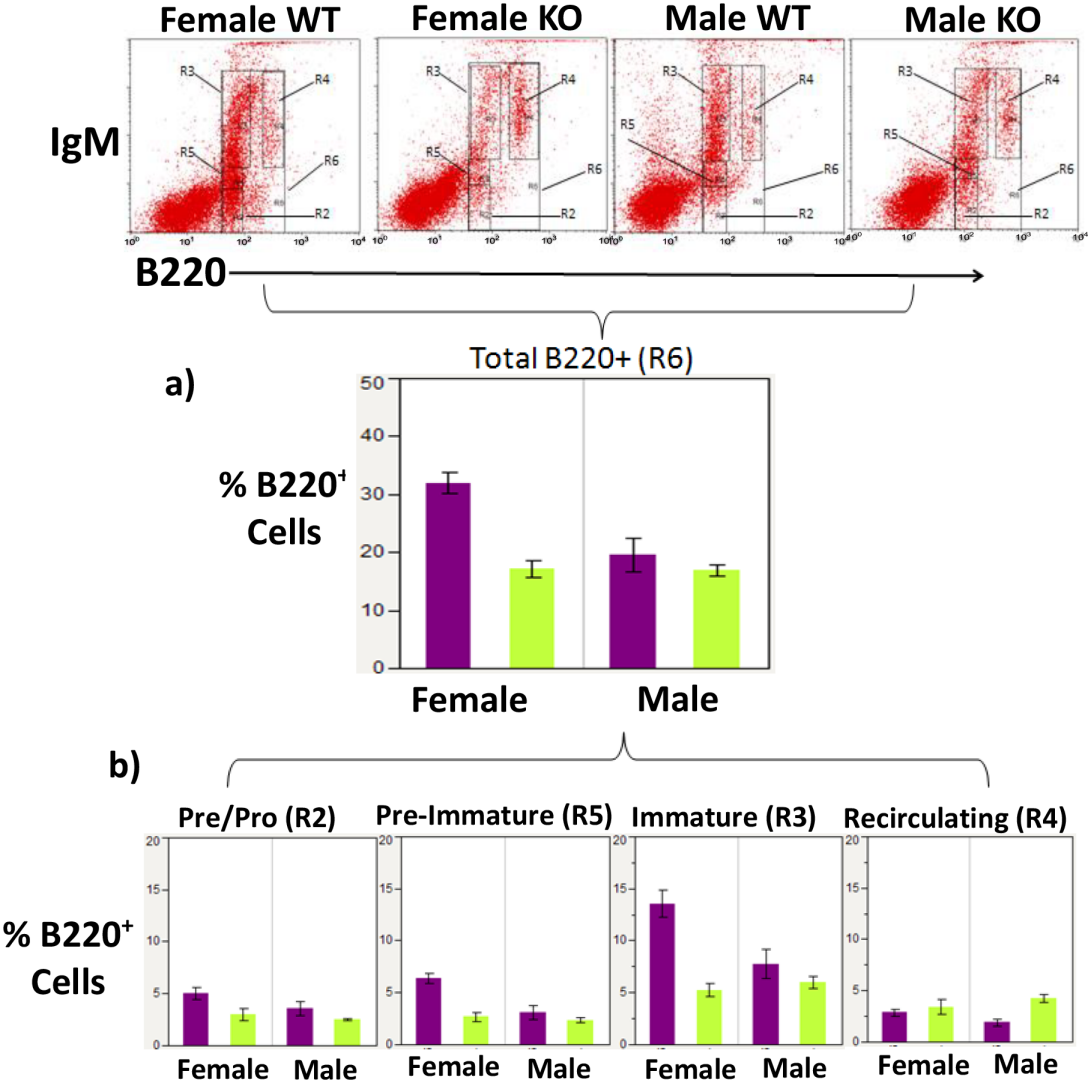
Development of B cells in the bone marrow of female and male Ly-6A/Sca-1 deficient mice. Bone marrow cells from female and male Ly-6A//Sca-1 and wild-type Ly-6A/Sca-1^+/+^ mice were gated based on their size and density to exclude dead cells (gating strategy shown in S1 Fig) and then analyzed for expression of B220 and IgM as shown in dot bots (upper panels). a) Percentage of developing B cells in the bone marrow of each genotype and sex, gauged by B220 (R6) expression is shown. The data shown is cumulative of at least 5 mice/genotype/gender. b) Percentage of living cells in the bone marrow at various stages of B cell development. Four different stages of B cell development were identified based on expression of B220 and IgM: IgM^-^ and B220^low^ Pre/Pro B cells (R2), IgM^high^ B220^low^ immature B cells (R3), IgM^high^ B220^high^ re-circulating B cells (R4) and a transitional stage expressing IgM^low^ B220^low^ between pre B cells and immature B cells (labeled pre-immature) (R5). Data presented as a percentage of live bone marrow cells from sex and genotype combinations. Data represents the mean with SEM. n = 5–7 per genotype/gender.

Ly-6A/Sca-1is one of several molecules expressed on HSCs, the early progenitor cells which give rise to all hematopoietic lineages [18] including B lymphocytes. Prior to assessing antibody responses generated in Ly-6A/Sca-1deficient mice in response to cOVA, we examined the development of B cell subsets in the bone marrow and other peripheral immune tissues in the Ly-6A/Sca-1 deficient mice. While examining B cell development in Ly-6A/Sca-1deficient mice we observed that female Ly-6A/Sca-1^-/-^ mice on a C57BL/6 background showed a 46% decrease in B220^+^ cells in the bone marrow when compared to female wild-type C57 BL/6 mice (p = 0.0003) (Fig 1a). This decrease was not observed in male Ly-6A/Sca-1^-/-^ mice when compared to control, age-matched male C57BL/6 mice (p = 0.81). To determine the stage at which the loss of Ly-6A/Sca-1affected B cell development we examined four developmental stages based on the expression of IgM and B220 proteins: Pre/Pro B cells (IgM^neg^B220^low^), immature B cells (IgM^high^ B220^low^), mature/re-circulating B cells (IgM^high^ B220^high^) and a transitional stage (IgM^low^ B220^low^), which we labeled pre-immature, FACS gating strategy for these subpopulations is shown in S1 Fig. At the pre-immature stage (IgM^low^B220^low^) of B cell development, the B220^+^ cells were decreased by 58%, and at the immature stage B220^+^ cells decreased by 61% in female Ly-6A/Sca-1^-/-^ bone marrow when compared to the bone marrow from female C57 BL/6 wild-type mice (p = 0.0004 and p = 0.0003 respectively) (Fig 1b). In contrast, we did not observe differences in these immature subsets of B cells in male Ly-6A/Sca-1^-/-^ mice when compared to male C57 BL/6 wild-type mice (p = 0.75 and p = 0.71 respectively) (Fig 1b). We did not observe a difference in the pre/pro B cell populations in males or females of wild-type and Ly-6A/Sca-1deficient mice (pre/pro B cells, female: p = 0.10; male: p = 0.57). Neither did we observe a difference within the mature/re-circulating B cell population between wild- type and Ly-6A/Sca-1deficient females (females, p = 0.78) (Fig 1b). However, we did observe difference in the mature/re-circulating B cells in the males (males, p = 0.007).

**Fig 2:**
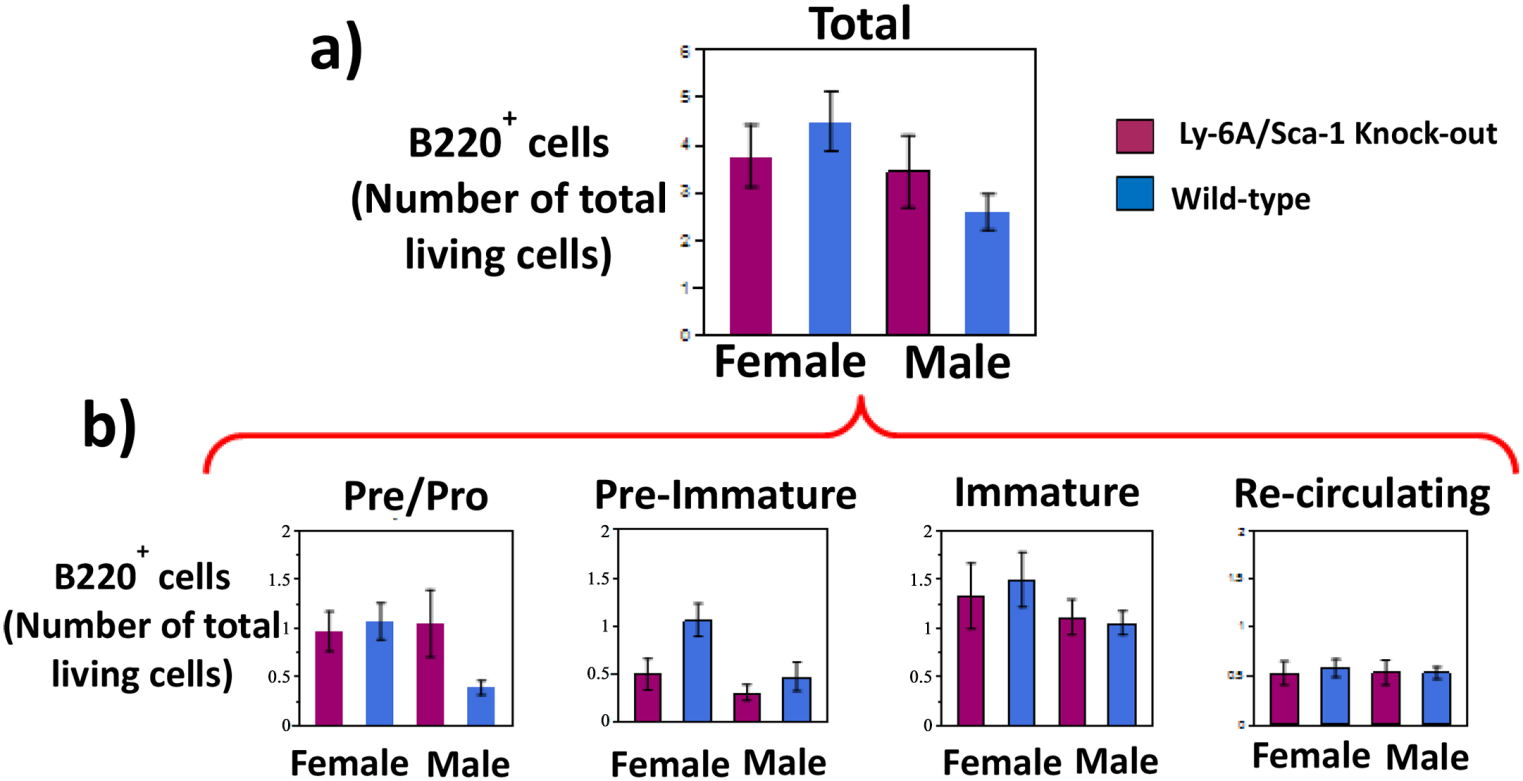
Absolute numbers of developing B cells in the bone marrow of female and male Ly-6A/Sca-1^-/-^ mice. Bone marrow cells from female and male Ly-6A/ Sca-1^-/-^ and wild-type Ly-6A/Sca-1^+/+^ mice were gated based on their size and density to exclude dead cells (gating strategy shown in S1 Fig) and then analyzed for expression of B220 and IgM (as in Fig 1). a) Total number (x10^6^) of developing B cells in the bone marrow of each genotype and gender, gauged by B220 expression is enumerated. The data shown is cumulative of at least 5 mice/genotype/gender. b) Total number of living cells in the bone marrow at various stages of B cell development. Four stages of B cell development were identified based on expression of B220 and IgM: IgM^-^ and B220^low^ Pre/Pro B cells (R2), IgM^high^ B220^low^ immature B cells (R3), IgM^high^ B220^high^ re-circulating B cells (R4) and a transitional stage expressing IgM^low^B220^low^ between pre B cells and immature B cells (labeled pre-immature) (R5). Data presented as a percentage of live bone marrow cells from sex and genotype combinations. Data represents the mean with SEM. n = 5–7 per treatment. Percentage representation of each of developmental subsets based on the expression of B220 and IgM was similar to what is presented in Fig 1.

While we observed sex-specific differences in the relative representation of developing B cell subsets, the absolute numbers of B cells produced in the bone marrow of Ly-6A/Sca-1^-/-^ mice was not significantly different from the wild-type (Fig 2), neither did we find any sex-specific differences as well (Fig 2). Furthermore, the absolute number within each of the developmental subset was not significantly different from the wild-type mice of the same gender (Fig 2).

Analysis of the B cell compartments in the spleen and lymph nodes of Ly-6A/Sca-1^-/-^ demonstrated unaltered representation of B cells when compare to Ly-6A/Sca-1 sufficient mice (S2 Fig) and numbers in spleen (data not shown). Taken together our data show that B cell development in male and female Ly-6A/Sca-1 deficient mice is similar to age and sex-matched wild-type littermates. However, we do observe sex-specific alterations in the percentage of B cells in the marrow of female Ly-6A/Sca-1 deficient mice that are likely cell non-autonomous.

We examined the expression of B220 and CD5 proteins to enumerate the B-1 and B-2 cells from the retrieved peritoneal exudates (PEC) from the wild- type and Ly-6A/Sca-1 Knockout mice [35]. Live lymphoid population were gated (S3 Fig) for enumeration of B-1a (CD5^Med^B220^Low^) and B-1b (CD5^-^B220^Low^) cells that constitute two subsets of B-1 cells in the peritoneum (Table 1). The data compiled in Table 1 analyzes B-1 cell subsets from young (5–8 weeks) and older (8.5–33 weeks), female (Table 1A & 1B) and male (Table 1C & 1D) mice of Ly-6A/Sca-1^-/-^ and wild-type genotype. Percentage of B-1a cells in the live lymphoid-gated population, examined across the gender and ages (Total mice analyzed = 43), ranged from 28–42% and 17–34% in the wild-type and Ly-6A/Sca-1^-/-^ mice, respectively. Representation of B-1a cells in the Ly-6A/Sca-1^-/-^ mice was not significantly different from the wild-type control when analyzed using two way ANOVA (p>0.05). B-1b cells in the live lymphoid gate ranged from 14.3–25.9% and 11.4–22.0% in the Ly-6A/Sca-1^-/-^ and wild-type mice, respectively, tested across gender and ages (n = 43). Two way ANOVA did not show a statistically significant difference between B-1b cells from Ly-6A/Sca-1^-/-^ and the wild-type control (p>0.05) of same gender and age group. As expected, statistically significant difference across the age and gender was observed when B-1a and B-1b subsets were analyzed (Table 1). In contrast, the percentage of B-2 cells present in the peritoneal exudates of young female Ly-6A/Sca-1 deficient mice was significantly higher than the age and sex-matched wild-type controls (32.5±2.3 Vs 17.6±1.5; p = 0.0001). Significant differences in the representation of B-2 subset was observed in both the young male Ly-6A/Sca-1 deficient mice when compared with the age and sex matched wild-type controls (25.8±3.3 Vs 14.4±3.3; p = 0.0001), as well. We did not observe statistically significant difference in the representation of peritoneal B-2 cells in older female Ly-6A/Sca-1 deficient mice when compared to the wild-type, age and sex-matched wild-type mice (34.5±2.3 Vs 33.1±3.3). T cells present in peritoneal washing, identified by their characteristic high expression of CD5 protein (CD5^High^ B220^-^) showed similar representation in the Ly-6A/Sca-1 deficient mice when compared to the age and sex-matched wild-type littermate controls (Table 1). While the percent representation of B-1a, B-1b, B-2 and T cells in peritoneum of the Ly-6A/Sca-1 knockout mice was significantly different from the wild-type littermates the absolute numbers were not. Taken together, our data shows that mice with Ly-6A/Sca-1 deficient mice did not show altered numbers of B1a and B1b cells in the peritoneal exudates compared to age and sex-matched wild-type controls. These data suggest that expression of Ly-6A/Sca-1 does not contribute to the development and/or homoeostasis of B-1 cell subsets. Differences in the relative proportions and not in the absolute numbers of B-2 cells in live lymphoid gated peritoneal exudates may suggest effects of Ly-6A/Sca-1 deficiency on either development, survival or trafficking of non-B and non-T lymphoid cell population(s) in the peritoneal exudates.

**Table 1:** B lymphocyte subsets in peritoneum of young (5–8 week) and older (8.5–33 week) old wild-type and Ly-6A/Sca-1^-/-^ mice.

**A**				
	**% Lymphocytes**^a^		**Lymphocyte numbers (x10**^**5**^**)**^b^	
**Phenotype**^c^	**Wild-type Young Female**	**Ly-6A/Sca-1**^**-/-**^**Young Female**	**Wild-type Young Female**	**Ly-6A/Sca-1**^**-/-**^**Young Female**
B-1a	39.9±3.3	31.1±3.7	1.7±0.4	0.7±0.5
B-1b	17.9±1.5	14.3±1.7	0.6±0.2	0.3±0.2
B2	17.6±2.1*	32.5±2.3*	0.7±0.7	0.6±0.7
T cells	17.8±2.4	19.4±2.7	0.6±0.4	0.4±0.4
Live lymphoid			3.8±1.7	2.0±1.9
Total cells			10.1±3.8	9.4±4.2
**B type and Ly-6A/Sca-1-/- mice**				
	**% Lymphocytes**^d^		**Lymphocyte numbers (x10**^**5**^**)**^e^	
**Phenotype**^f^	**Wild-type Older Female**	**Ly-6A/Sca-1**^**-/-**^**Older Female**	**Wild-type Older Female**	**Ly-6A/Sca-1**^**-/-**^**Older Female**
B-1a	28.2±5.3	29.9±3.7	2.8±0.7	1.9±0.5
B-1b	11.4±2.4	17.0±1.7	1.2±0.3	1.1±0.2
B2	33.1±3.3	34.5±2.3	3.2±1.0	3.1±0.7
T cells	25.0±3.8	15.7±2.7	2.3±0.6	1.6±0.4
Live lymphoid			9.6±2.6	8.1±1.9
Total cells			23.3±5.9	24.7±4.2
**C**				
	**% Lymphocytes**^g^		**Lymphocyte numbers (x10**^**5**^**)**^h^	
**Phenotype**^i^	**Wild-type Young male**	**Ly-6A/Sca-1**^**-/-**^**Young male**	**Wild-type Young male**	**Ly-6A/Sca-1**^**-/-**^**Young male**
B-1a	42.6±5.3	34.4±5.3	0.2±0.7	0.2±0.7
B-1b	22±2.4	17.0±2.4	0.1±0.3	0.1±0.3
B2	14.4±3.3*	25.8±3.3*	0.1±1.0	0.14±1.0
T cells	13.4±3.8	24.1±3.8	0.1±0.6	0.1±0.6
Live lymphoid			0.5±2.6	0.6±2.6
Total cells			2.9±5.9	2.5±5.9
**D**				
	**% Lymphocytes**^j^		**Lymphocyte numbers (x10^5^)**^k^	
**Phenotype**^l^	**Wild-type Older male**	**Ly-6A/Sca-1**^**-/-**^**Older male**	**Wild-type Older male**	**Ly-6A/Sca-1**^**-/-**^**Older male**
B-1a	32.4	16.6±5.3	1.1	0.5±0.7
B-1b	13.9	25.9±2.4	0.5	1.2±0.3
B2	17.4	33.3±3.3	0.6	1.0±1.0
T cells	30.3	16.4±3.8	1.0	0.6±0.6
Live lymphoid			3.3	3.2±2.6
Total cells			7.5	15.3±5.9

^a,d,g,j^ Percent least square mean (±SEM) of lymphocytes based live lymphocyte gate based on forward and side scatter pattern, myeloid/granulocyte/erythroid populations and dead cells were excluded from this gate.

^b,e,h,k^ Least square mean B cell numbers (±SEM) were calculated based on total cells harvested from peritoneal cavity using JMP statistical software.

^c,f,i,l^ Phenotype of B-1 and B-2 B cell subsets were identified based on the expression of CD5 and B220. B1a: CD5^Med^B220^Low^; B-1b: CD5^-^B220^Low^; B2: CD5^-^B220^High^; T cells: CD5^High^B220^-^.

* The percentage or number of cells significantly different from the wild-type mice, *p* = 0.0001. Total number of mice used, n = 43; Wild-type (10 young females, 4 old females, 4 young males, 1 old male; Ly-6A/Sca-1^-/-^ (7 young females, 9 older females, 4 young males and 4 old males). Statistical significance reported here was from only those groups with 4 or mice in each genotype/age cohort.

Effective antibody responses generated by B cells are dependent on CD4^+^ helper T lymphocytes [16, 17] but the development of CD4^+^ T cells in Ly-6A/Sca-1 deficient mice has not been fully examined. To investigate alteration in the early T cell development, we examined the expression of CD44 and CD25on the triple negative (CD4^-^CD8^-^CD3^-^) T cells in the thymus; this early developmental subset constitutes up to 5.2%–8.6% of total live cells in the thymus. We did not observe any significant difference between Ly-6A/Sca-1^-/-^ and the wild-type mice in either sex, at any of the four earliest stages of T cell development (S4 Fig) (CD25^+^ CD44^-^ female: p = 0.26 male: p = 0.94. CD25^+^ CD44^+^ female: p = 0.76 male: p = 0.82. CD25^-^ CD44^+^ female: p = 0.84 male: p = 0.41. CD25^-^ CD44^-^ female: p = 0.90 male: p = 0.61). In addition, we did not observe any alterations in the development of CD4^+^CD8^+^ immature and mature helper and cytotoxic T cells in the thymus of Ly-6A/Sca-1mice when compared to the age and sex-matched wild-type littermates (S4 Fig) (CD4^-^ CD8^-^ female: p = 0.93 male: p = 0.41. CD4^+^ CD8^+^ female: p = 0.77 male: p = 0.10. CD4^+^ CD8^-^ female: p = 0.78 male: p = 0.13. CD4^-^ CD8^+^ female: p = 0.41 male: p = 0.99). Taken together, these data indicate that the absence of Ly-6A/Sca-1 does not affect early and late T cell development in the thymus.

### Primary antibody response to chicken ovalbumin in Ly-6A/Sca-1 deficient mice

**Fig 3:**
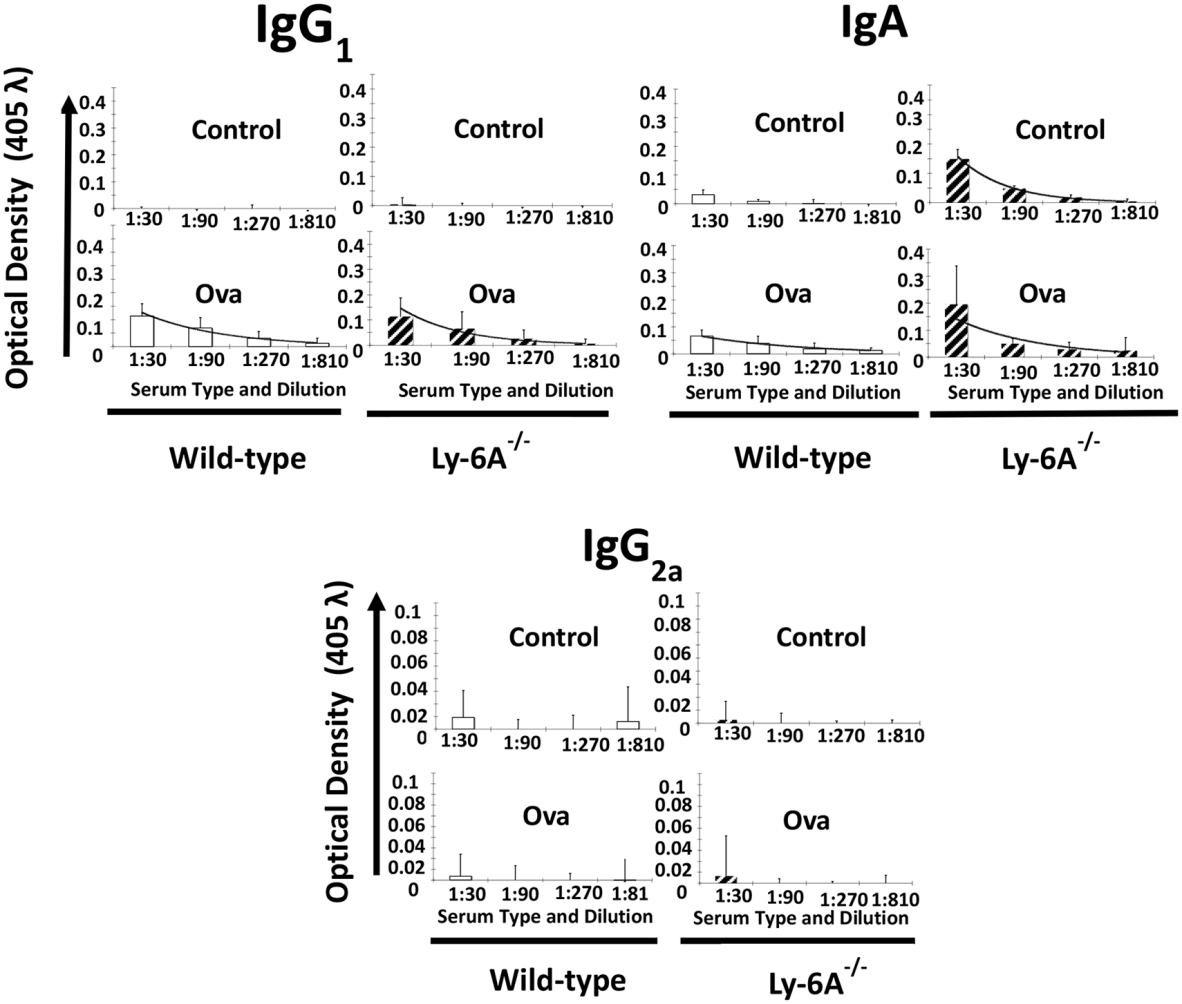
Anti-cOVA IgG1, IgA and IgG2a antibody response generated in Ly-6A/Sca-1^-/-^and wild- type female mice. Wild-type, Ly-6A/Sca-1^+/+^ (open bars) and Ly-6A/Sca-1knockout (hatched) animals were injected with TiterMax Gold (control), 100 μg OVA mixed with TiterMax Gold (Ova). Serum from blood drawn from the immunized mice on day-21 post-immunization (4 animals/genotype/experimental group) was examined for anti-cOVA antibody of IgG1, IgA and IgG2a isotype antibodies by ELISA. Each sample was tested in duplicate. Data were pooled, and graphs display average optical density (OD) with standard deviation of all samples (n = 4 per group, per genotype).

To investigate the effects of Ly-6A/Sca-1expression on functional primary B cell responses, female Ly-6A/Sca-1^-/-^ and wild-type mice were each injected with 100 μg of chicken ovalbumin (cOVA) protein antigen mixed with adjuvant TitreMax, or with adjuvant alone as a control. The serum of injected animals was tested for the presence of anti-OVA antibodies of various isotypes using ELISA. Ly-6A/Sca-1^-/-^ and wild-type mice injected with the adjuvant, TiterMax alone failed to generate anti-OVA IgG1 antibodies as expected (Fig 3). In contrast, anti-OVA IgG1 antibodies were generated at comparable levels in Ly-6A/Sca-1^-/-^ and wild-type mice that received c-OVA along with the TiterMax adjuvant. Kruskal-Wallis analysis of the data indicated no significant difference between adjuvant-alone injected control groups, and cOVA injected groups. In contrast to IgG1, we were either unable to detect (IgG2a isotype) or detect at low levels (IgA isotypes) other isotypes of anti-cOVA antibodies in cOVA antigen with adjuvant or adjuvant-alone injected wild-type mice (Fig 3). However, we observed the presence of higher levels of anti-OVA IgA antibodies in the serum from Ly-6A/Sca-1^-/-^ than the wild- type mice injected with cOVA antigen and adjuvant (Fig 3). Surprisingly, Ly-6A/Sca-1^-/-^ mice injected with cOVA with adjuvant had comparable levels of anti-OVA IgA antibodies as compared to those injected with adjuvant alone. These data suggest non-specific binding of IgA antibodies from Ly-6A/Sca-1^-/-^ mice to cOVA coated ELISA plates. This non-specificity was significantly lower when serum from wild-type littermate controls was used (Fig 3). Kruskal-Wallis analysis indicated that within each genotype there was no difference between adjuvant injected controls and cOVA with adjuvant injected groups. Taken together, our data suggests that primary antibody responses generated in Ly-6A/Sca-1^-/-^ mice are not significantly different from those generated in wild-type control mice. In addition, we detected higher levels of IgA in Ly-6A/Sca-1^-/-^ mice injected with adjuvant alone, with non-specific binding property.

### Elevated IgA λ levels in Ly-6A/Sca-1 knockout mice

**Fig 4:**
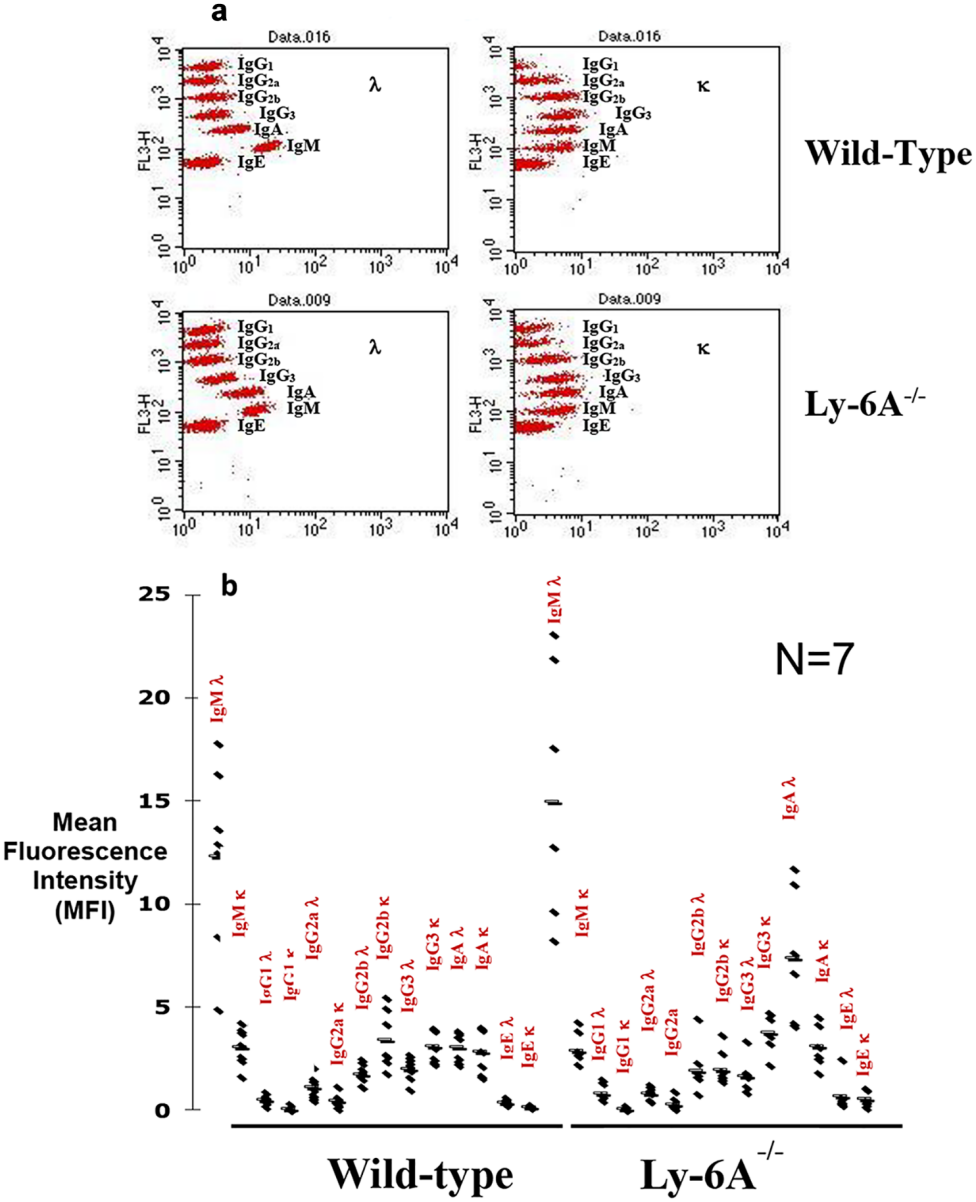
Analysis of serum immunoglobulin isotypes. Serum from Ly-6A/Sca-1^-/-^ and wild-type (WT) female mice was analyzed via Cytometric Bead Array that specifically detect immunoglobulin heavy and light (κ & λ) chains by flow cytometer. A representative bead array dot blot is shown in panel a. Immunoglobulin isotypes with the κ or λ light chain were detected with both the isotype and light chain- specific beads for semi-quantitative analysis using mean fluorescence intensity (MFI). Each dot represents one individual mouse per genotype (b). Seven mice were analyzed per genotype. * denotes statistical significance (IgA λ p = 0.003; IgG2b κ p = 0.04).

High non-specific binding of IgA antibodies in cOVA with adjuvant or adjuvant alone injected Ly-6A/Sca-1mice was intriguing and therefore prompted us to examine the basal levels of all the isotypes in Ly-6A/Sca-1^-/-^ and wild-type mice. We used cytometric bead array (CBA) to examine the basal levels of serum immunoglobulin in Ly-6A/Sca-1^-/-^ mice. While both the wild-type and Ly-6A/Sca-1^-/-^ knockout animals had high levels of serum IgM, Ly-6A/Sca-1 knockout animals had significantly higher levels of serum IgA λ than their wild-type control littermates (Fig 4). IgM, IgG_1_, IgG_2a_, IgG_3_, and IgE κ and λ chains, as well as IgA κ and IgG2b λ isotypes were not significantly altered in the Ly-6A/Sca-1deficient mice when compared to the littermate controls (Fig 4).

### Unaltered receptor editing in B cells from Ly-6A/Sca-1^-/-^ mice

**Fig 5:**
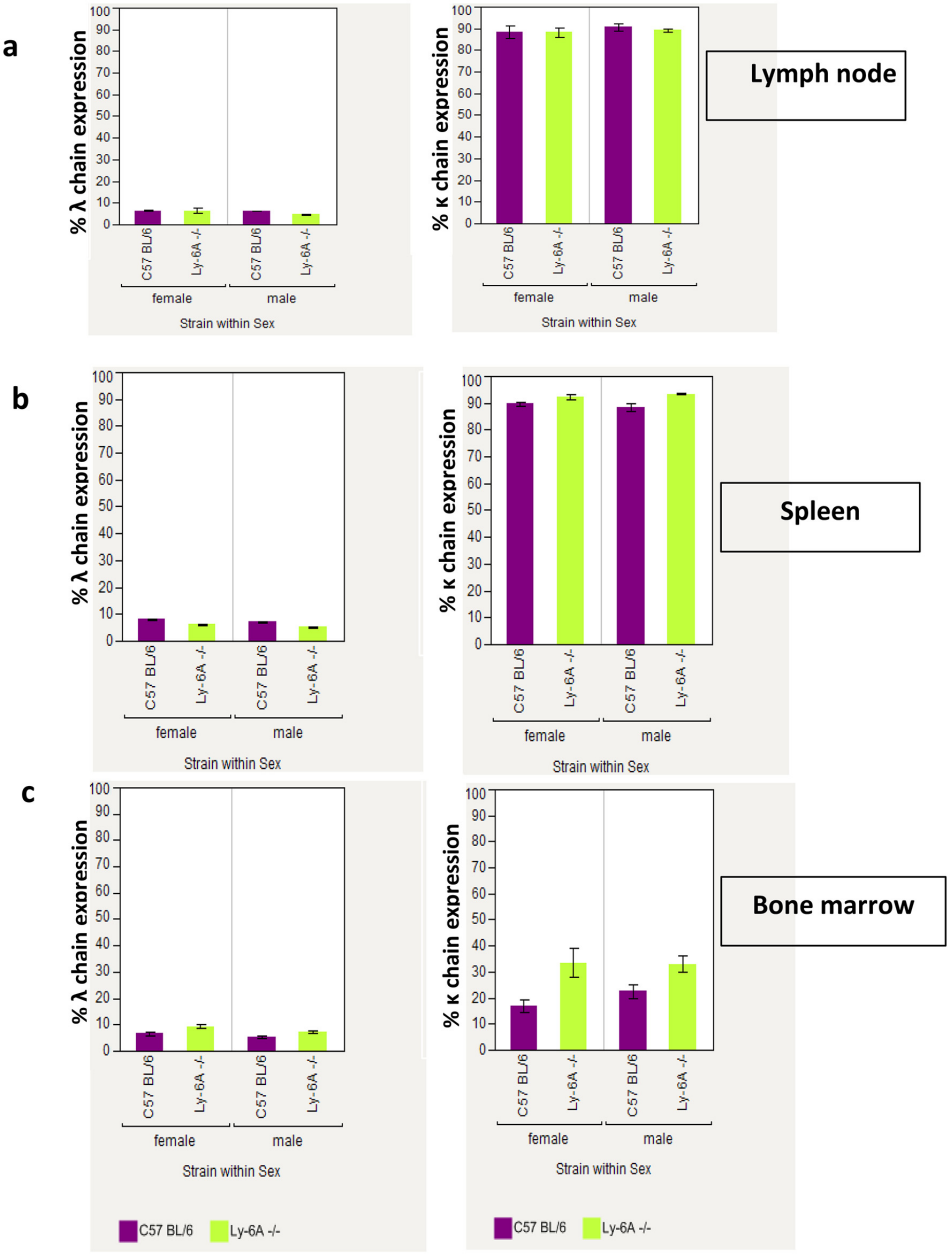
Flow cytometry analysis of immunoglobulin light chain expression on B220^+^ cells in the lymph node, spleen and bone marrow of Ly-6A/Sca-1^-/-^ and Ly-6A/Sca-1^+/+^ wild-type mice. a). Percentage of λ and κ light chain positive cells within the B220^+^ cells from the lymph node is shown. b). Percentage of λ and κ light chain positive cells within the B220+ cells from the spleen is shown. c). Percent λ and κ light chain positive cells within the B220^+^ cells from the bone marrow is shown. Data represents the mean with SEM. n = 4–5 mice per genotype/gender.

Developing B cells in the bone marrow show high representation of λ light chain due to a process known as “receptor editing” [36, 37]. Higher level of serum IgA λ detected in the Ly-6A/Sca-1deficient mice than the wild-type littermates (Fig 4) was surprising. Therefore, we determined the immunoglobulin light chain usage in developing and mature B cells present in the bone marrow and peripheral lymphoid tissues of Ly-6A/Sca-1deficient mice (Fig 5, Table 2 and S5 Fig). Similar κ and λ light chain usage was detected in Ly-6A/Sca-1^-/-^ mice as the wild-type littermates (Fig 5).

**Table 2:** λ and κ light chain expression on B lymphocyte in Peyer’s patch of 5–8 week old wild-type and Ly-6A/Sca-1^-/-^ mice.

Phenotype^a^	% Lymphocytes^b^		Lymphocyte numbers (x10^4^)^c^	
	Wild-type	Ly-6A/Sca-1^-/-^	Wild-type	Ly-6A/Sca-1^-/-^
IgA^+^ λ^+^	3.8*	3.5+/-0.8	2.9*	1.4+/-0.6**
IgA^+^ κ ^+^	89.0*	90.6+/-6.9	67.0*	37.3+/-13.3
IgD^+^ λ^+^	4.1+/-1.6	3.7+/-1.5	3.4+/-1.5	2.8+/-2.0
IgD^+^ κ ^+^	89.7+/-9.0	86.2+/-1.0	79.0+/-15.1	48.7+/-15.5
B220^+^ λ^+^	3.0+/-2.5	3.6+/-2.2	2.2+/-1.7	1.5+/-0.6
B220^+^ κ ^+^	89.5+/-4.7	82.8+/-6.2	87.3+/-18.5	29.0+/-30.1***

^a^ Phenotype of B cell subsets were identified based on the expression of surface IgA, IgD and B220. Each of the above subset was examined for the expression of either λ of κ light chain. λ^high^ and κ^Intermed-high^ expressing cells were enumerated. Total number of mice used, n = 23; Wild-type, n = 6 (3 females, 3 males); Ly-6A/Sca-1^-/-^ n = 17 (13 females, 4 males). No significant differences between the male and female mice within a genotype was observed (data not shown), therefore combined data is shown here.

^b^ Percent lymphocytes based on forward and side scatter pattern of live lymphocyte gated cells, myeloid/granulocyte/erythroid populations and dead cells were excluded from this gate. No Statistical significance across genotype with combined sex cohorts was observed.

^c^ Mean numbers of B cell subset (±SEM) were calculated based on total cells harvested from peyer’s patch/mouse. *P* values were calculated by two-tailed test.

* Average percentage and number of cells/mouse reported were from pooled peyer’s patch from three mice before FACS analyses.

**p = 0.002;

***p = 0.047.

**Fig 6:**
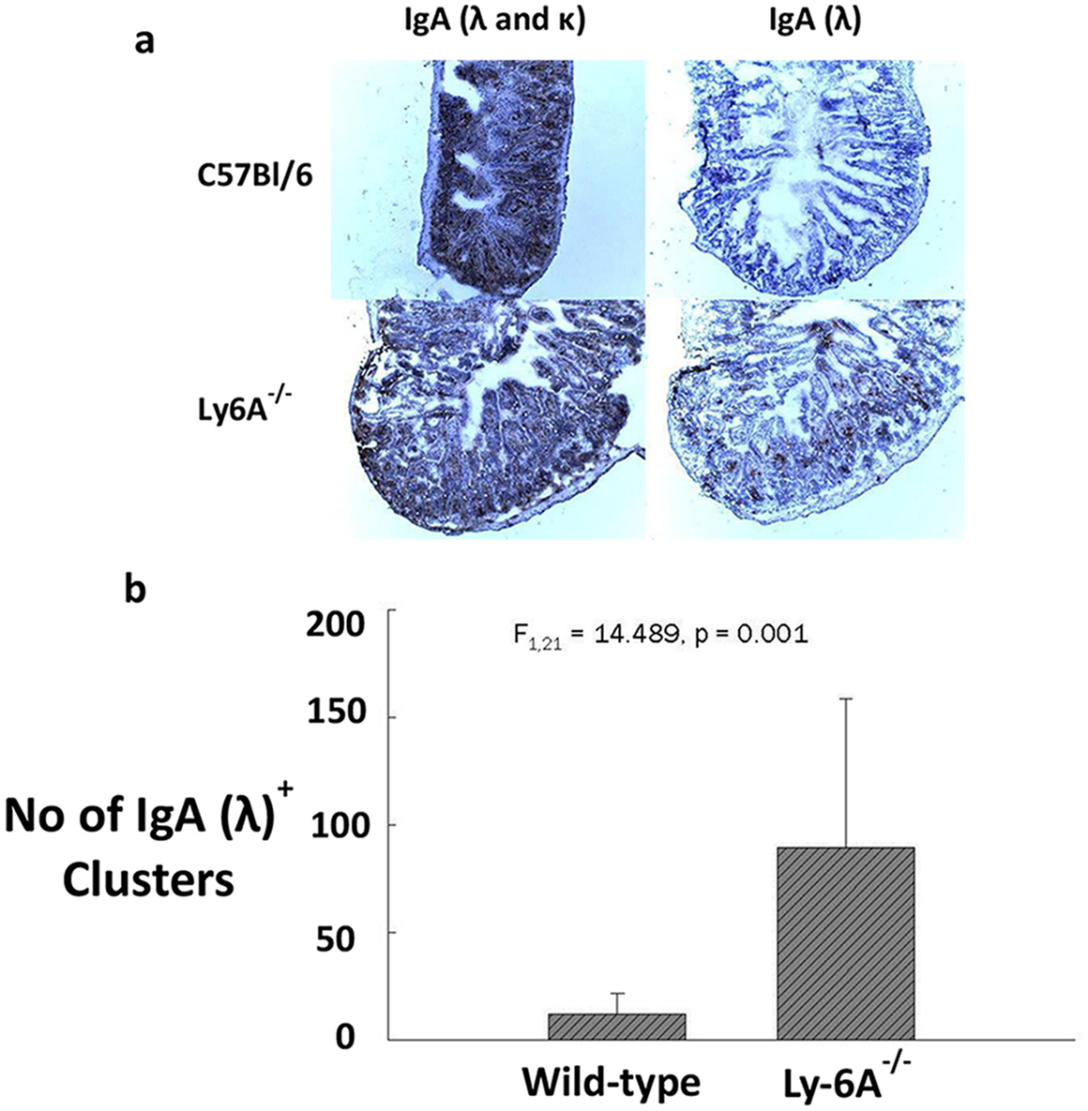
Detection of IgA (λ & κ) and λ light chain lamina propria of Ly-6A/Sca-1^-/-^ and wild-type C57bl/6 mice. Ileum from the wild-type and Ly-6A/Sca-1^-/-^ mice were cut with a microtome to obtain 5–8 μm thick sections which were then stained with biotinylated anti-IgA and anti-Ig λ primary antibodies followed by ABC (anti-biotin-biotin complex) kit from Vector Laboratories. Sections were then stained with H_2_O_2_ and chromogen Di-Amino Benzedine (DAB) followed by counterstaining with hematoxylin QS (Vector Laboratories). Images of ileum tissue sections were taken at 100X magnification. a) A representative staining for the wild-type and Ly-6A/Sca-1^-/-^ mice is shown. b) Numbers of IgA λ^+^ light chain clusters. Clusters of cells stained within the ileum tissue sections were counted and data was represented as the average number of clusters from four mice/genotype. One way ANOVA was used to determine statistical significance among the groups (n = 22, F_1,21_ = 14.489, *p* = 0.001). Error bars were taken across all tests.

However, the absolute numbers of λ and κ expressing IgA^+^, or IgD^+^ or B220^+^ B cells in peyer’s patch (PP) of Ly-6A/Sca-1^-/-^ mice appeared to be reduced when compared to the wild-type control mice, the significant differences were only observed in IgA^+^λ^+^ (p = 0.002) and B220^+^κ^+^ (p = 0.047) subsets (Table 2 and S5 Fig). In contrast to these findings, we detected high expression of λ light chain on clusters of B cells in LP of Ly-6A/Sca-1^-/-^ mice (Fig 6). These findings indicate that B cells in LP uniquely produce high levels of IgA with λ light chain. Normal representation of λ light chain expressing B cells in the bone marrow, spleen, lymph node and PP of Ly-6A/Sca-1^-/-^ mice with altered detection only in LP suggests that the B cells in the LP is the source of high levels of IgA λ in the serum of Ly-6A/Sca-1^-/-^ mice.

## Discussion

Antibody responses generated by B cells are critical for humoral immunity against pathogens. For an effective T-dependent response B lymphocytes garner help from CD4^+^ helper T cells. A number of proteins expressed on either naïve or activated helper T cells contribute to cognate interactions with B cells by binding to their ligands [38, 39]. Cytokines secreted by helper T cells are known to contribute to this “helping function” [40]. Ly-6A/Sca-1 is constitutively expressed on 35% of primary CD4^+^ T cells and its expression is up-regulated >100 fold upon activation of CD4^+^ T cells [7]. Contribution of Ly-6A/Sca-1expression on CD4^+^ T cells to primary antibody response to a foreign antigen is not known. Our findings suggest that the up-regulated expression of Ly-6A/Sca-1does not contribute to primary antibody responses in vivo (Fig 4). To our surprise the Ly-6A/Sca-1^-/-^ mice show altered expression of λ light chain in LP of gut associated lymphoid tissue (Fig 6).

T-dependent B cell responses can be viewed to occur in three distinct phases (reviewed in [17]). The first phase involves generation of effector helper T cells expressing CD40L, other regulatory proteins and chemokine receptors. The second phase involves cognate interaction between effector T cells with antigen-primed B cells. This phase involves trafficking of antigen-experienced B and effector helper T cells towards the junction of B and T cell zones in the secondary lymphoid tissues and antigen presentation where by B cells present the captured antigens to effector helper T cells. In addition, among other receptor-ligand interactions, the binding between CD40 on B cells with CD40L on effector T cells provides co-stimulation. The third phase involves germinal center reaction and development of generation of short and long-term memory B cells and differentiation into plasma cells outside the germinal center. The first and second phase of T-dependent B cell response occurs within 7 days of antigen exposure whereas the third phase memory B cells and plasma cells are generated 7 days after antigen exposure [17]. Ly-6A/Sca-1 expressed on primary CD4^+^ T cells at low levels and activated T cell at higher levels can potentially contribute to B cell responses at different phases of B cell response. Diminished secondary antibody responses to an antigen KLH were reported in Ly-6A/Sca-1 deficient mice [11]. How Ly-6A/Sca-1 contributes to secondary antibody response after antigen challenge and insignificantly participates in the primary antibody response remains unclear. It is formally possible that early expression of Ly-6A/Sca-1 on the first round of activated CD4^+^ population after primary immunization is not optimal in generating effector/long-lasting plasma cells during the primary antibody response. We speculate that a higher in vivo expression of Ly-6A/Sca-1 on CD4^+^ T cells, which is achieved after re-challenge with the antigen, is required for generating a long lasting plasma/memory cell population and/or their survival/trafficking to bone marrow and higher antibody responses. Ly-6A/Sca-1and other Ly-6 genes possess interferon-responsive elements [15] and expression of Ly-6A/Sca-1 on activated CD4^+^ T cells is driven by the action of interferon-γ [14]. The source of interferon-γ and pharmacokinetics of this cytokine required to drive in vivo expression of Ly-6A/Sca-1 remains unknown, but it is higher after antigen challenge than during the primary exposure with the antigen. This is consistent with the idea that naïve CD4^+^ T cells generate relatively less interferon-γ cytokine than the effector and memory T cells [41, 42]. Alternatively, it remains possible that, early on, the role of Ly-6A/Sca-1 is redundant and substituted by another Ly-6 family member. Ly-6C, another member of the Ly-6 family, is reported to have a role in B cell responses. CD4^+^ T cells with high expression of Ly-6C express distinct repertoire of TCRs that promote plasma cell production [43]. Additional work will be required to address the role of Ly-6A/Sca-1 in secondary/memory cell responses, our observations provide an impetus to examine the role of Ly-6A/Sca- Ly-6A/Sca-1 expression during differentiation phases of T-dependent B cell responses and antibody generation.

In addition to its expression on naïve and activated T cells, Ly-6A/Sca-1 shows expression on progenitor cells and its differentiated states [1, 2]. While Ly-6A/Sca-1 deficiency results in diminished development of megakaryocytes [33], its role of Ly-6A/Sca-1 in the development of B and T cell lineages has not been fully described. Given the expression of Ly-6A/Sca-1 on HSCs as well as on early lymphoid progenitors that give rise to B and T lymphocytes it was important to study the effects of Ly-6A/Sca-1 on the development of the lymphoid compartment. Ly-6A/Sca-1 deficient mice neither show significantly altered numbers of B-1a, B1b subsets in the peritoneum nor exhibit effects on development of mature follicular B cells in the bone marrow, spleen and lymph nodes (Table 1). In addition, Ly-6A/Sca-1 deficient and sufficient mice show similar development of T cells in the thymus. While the absolute number of B cell and T cell subsets in the Ly-6A/Sca-1 deficient and Ly-6A/Sca-1 sufficient mice were similar (Fig 2), we did observe altered representation (percentages) of B-2 cell subsets in the peritoneum and developing B cells in the bone marrow. Some of these differences were sex-specific. While female C57BL/6 wild-type mice have a higher representation of B220^+^ cells in the bone marrow than male wild-type mice, the female C57BL/6 mice with a deficiency in the Ly-6A/Sca-1gene show significantly diminished representation of B220^+^ cells in the bone marrow as compared to Ly-6A/Sca-1 sufficient control mice of the same sex and strain. The developing B cells of female Ly-6A/Sca-1 deficient mice have a similar profile to that of male mice, both wild-type and Ly-6A/Sca-1^-/-^ (Fig 1). The female Ly-6A/Sca-1 deficient mice showed altered representation of some developing B cell subsets in the bone marrow (IgM^high^ B220^low^ immature B cells and IgM^low^B220^low^ transitional subset), the representation of other developing B cell subsets in the bone marrow was not significantly altered (IgM^-^B220^low^ pre/pro B cells) when compared to female, wild-type controls. In addition, mature B cell population in the lymph node and spleen of Ly-6A/Sca-1^-/-^ mice was unaltered in the Ly-6A/Sca-1^-/-^ mice when compared to the wild-type (S2 Fig). Taken together these findings suggest that Ly-6A/Sca-1 does not influence development of B cells. Previous report on Ly-6A/Sca-1 null mice show subtle differences in total numbers and percentages of CFUs for some lineages which were deemed statistically insignificant [11, 33] but sex-specific differences were not evaluated in these reports. Sex-specific altered proportions of B cell subsets that we have observed in the bone marrow suggest that Ly-6A/Sca-1 influences development of yet unknown immune cell population within the lymphoid population, identity which will requires further investigation. Future investigations will also require to fully examine the normal expression of Ly-6A/Sca-1 on CLPs and other progenitors and how its absence alters their numbers and quality in either of the sexes.

Regardless of the genotype, we observed that female mice showed distinct representation of B cells (Fig 1). Published reports show that hormones in itself or in combination with sex-linked gene products affect B cell development in the bone marrow and the development of T cells in the thymus [44–45]. Precursors of immune cells, as well as their progeny, express receptors for sex-specific steroid hormones at different development stages [46]. Androgens affect B cell development through androgen receptors expressed on bone marrow stromal cells [47], and while estrogens can exert their effects by signaling through intracellular estrogen receptors expressed in B and T cells [44, 45, 48]. Moreover, there is a considerable published studies suggesting that males and female mice mount distinct immune responses to self and foreign antigens and that these differences may be crucial to a sex-bias observed during a severe infection [48] and autoimmunity [49].

We examined the T cell development in Ly-6A/Sca-1^-/-^ mice starting from the CD44^+^CD25^-^CD4^-^ CD8^-^ CD3^-^ stage of their development in both male and female mice. The CLPs/ELPs, and their earliest progeny in the thymus marked by their expression or lack of expression of CD25 and CD44 also express Ly-6A/Sca-1 [34]. Ly-6A/Sca-1 is expressed during stage I (CD3^-^CD4^-^CD8^-^CD44^+^CD25^-^) and stage II (CD4^-^CD8^-^CD3^-^CD44^+^CD25^+^) of developing T cells in the thymus. This expression is turned off at stage III (CD3^-^CD4^-^CD8^-^CD44^-^CD25^+^) and stage IV (CD3^-^CD4^-^CD8^-^CD44^-^CD25^+^) of developing thymocytes. Data presented here suggest that the expression of Ly-6A/Sca-1 on stage I and stage II of TN cells does not play a role in the developmental progression to stage III of TN cells (S4 Fig). While the absence of Ly-6/Sca-1 does not affect early T cell development, down-regulation of normal expression of Ly-6A/Sca-1 expression on stage III of TN thymocytes is necessary for normal T cell development [34]. Our data is consistent with a previous investigation using these mice where Ly-6A/Sca-1 deficient mice were reported to have normal development of TCRαβ, TCRγδ, CD4^+^, CD8^+^ T cells [11]. Data presented here extends these studies to both the sexes and also focuses on early developmental stages where Ly-6A/Sca-1 expression is normal expressed. More importantly normal development of CD4^+^ T cells in Ly-6A/Sca-1 deficient mice implies that primary immune response to a foreign protein, cOvalbumin is not influenced by the numbers of CD4^+^ T cells in these mice when compared to the wild-type controls.

Interestingly, Ly-6A/Sca-1 deficient mice exhibited unusually higher representation of λ light chain than the wild-type littermates when their serum and LP was examined. Higher representation of λ light chain was exclusively observed in association with α heavy chain and not with μ, γ1, γ2a, γ2b, ε heavy chains in the serum. To investigate the source of circulating IgA with λ light chain prompted us to examine the expression of λ light chain on developing B lymphocytes in the bone marrow and in secondary lymphoid tissues. To examine the mechanism we speculated that this high natural circulating IgA λ may be derived from B cells undergoing excessive receptor editing, where switch from κ to λ light chain occurs in the bone marrow to generate a new B cell receptor to potentially avoid self-reactivity [36, 37]. Contrary to this notion, representation of κ and λ light chains expressed on developing and mature B cells in the bone marrow lymph nodes and peyer’s patch of Ly-6A/Sca-1 deficient mice was unaltered and therefore ruling out the contribution of Ly-6A/Sca-1 in the process of receptor editing during B cell selection in the bone marrow. In addition, by using immuno-histological techniques we were able to observe high frequency of λ light chain expression in the lamina propria of Ly-6A/Sca-1 deficient mice. However, higher expression of λ light chain from B cell clusters detected by immunohistochemistry suggests that Ly-6A/Sca-1 expression in the lamina propria influence light chain usage. While previous investigations have revealed that lamina propria has high number of IgA producing plasma cells with hallmarks of IgA switch recombination [50, 51], the mechanism underlying preferential usage of λ light chain by IgA^+^ B cells is not known. One possible mechanism may involve CD4^+^ T cells and cytokines they produce in driving this process. Previous reports show altered proliferation and cytokine profile in primary CD4^+^ T cells with altered expression of Ly-6A [12]. Additionally, other investigators have reported the effects of Ly-6A expression on signaling through TGF-βR1/II [52] and effect of TGF-β on suppressing the expression of Ly-6A (Sca-1) [53] on non-lymphoid cells. These published data provide a possible mechanistic framework for future experiments that we hope will examine a direct or indirect influence of Ly-6A expression on immunoglobulin light chain usage in the gut. Alternate possible mechanism may involve contribution of non-CD4^+^ T cells (dendritic or epithelial cells) and gut flora in this process. LYPD8, another member of Ly-6 supergene family is known to bind component of gut flora to prevent their proximity to the barrier surface in the gut [54]. Absence of Ly-6A/Sca-1 may provide an altered microenvironment conducive for either the self of gut microflora antigens to exert influence on B cells. Factors that influence light chain switching in LP are unknown and to our knowledge this is the first example where higher representation of λ light chain is reported in LP molecular mechanism of which requires future investigation.

## Supporting Information

S1 Fig Gating strategy for Flow cytometric analyses of B cells in the bone marrow of Ly-6A/Sca-1^-/-^ and Ly-6A/Sca-1^+/+^ wild-type mice. Cells were harvested from the bone marrow as described in materials and methods spleen and stained with anti-B220-FITC and anti-IgM-PE and analyzed by flow cytometer. Live lymphoid population (left panel, R1) was gated and examined for surface expression of B220 and IgM (right panel). Data with this gating strategy is shown in Figs 1 and 2.
(TIF)

S2 Fig Flow cytometric analyses of B220^+^ cells in the lymph node and spleen of Ly-6A/Sca-1^-/-^ and Ly-6A/Sca-1^+/+^ wild-type mice. Cells were removed from the lymph nodes and spleen and stained with anti-B220 and anti-CD3ε (panels A & B) or anti-IgD and anti-IgM antibodies (panels C & D). Dot-blots for staining of live lymphoid gated spleen cells is shown (panels A-D). Cumulative data from lymph node and spleen cells is presented as a percentage of B220^+^ cells from gender and genotype combinations. Data represents the mean with SEM. n = 5–9 mice per genotype or sex.
(TIF)

S3 Fig Flow cytometry gating strategy for analysis of B-1 B cells in the peritoneum of wild-type and Ly-6A/Sca-1^-/-^ mice. Live lymphocytes (R1) were gated based on forward and side scatter pattern (myeloid/granulocyte/ erythroid populations and dead cells were excluded) (panel A) and B-1 and B-2 B cell subsets were identified based on the expression of CD5 and B220 (panel B). B1a: CD5^Med^B220^Low^ (R3); B-1b: CD5-B220^Low^ (R5); B2: CD5-B220^High^ (R4); T cells: CD5^High^B220^-^ (R2). Data analyses is shown in Table 1 of the manuscript.
(TIF)

S4 Fig Flow cytometry analysis of developing T lymphocytes in the thymus of Ly-6A/Sca-1^-/-^ and Ly-6A/Sca-1^+/+^ wild-type mice. Live lymphocytes (R1) were gated based on forward and side scatter pattern (excluding dead cells) (R1 gate in panel A) and stained with anti-CD3ε, anti-CD4 and anti-CD8 (all three conjugated with same fluorophore) along with anti-CD44 and anti-CD25. The triple negative T cells (CD4^-^CD8^-^ CD3^-^) (R2 gate in panel B) at four distinct stages of early T cell development based on the expression of CD44 and CD25 are shown (panel C). Analysis of helper and cytotoxic T cells in the thymus of Ly-6A/Sca-1 ^-/-^ mice. Percentage of living thymocytes at four distinct stages of late T cell development based on the expression of CD4 and CD8 proteins (panel D). Data is presented as a percentage of living thymocytes from sex and genotype combinations. Data represents the mean with SEM. n = 4–5 per genotype/sex.
(TIF)

S5 Fig Gating strategy for the analyses of on κ and λ light chain expression on B220^+^ cells in the bone marrow of Ly-6A/Sca-1^-/-^ and Ly-6A/Sca-1^+/+^ wild-type mice. A). Gating strategy for κ light chain expressing B cells. Live cells (R1 gate) were gated based on forward and side scatter pattern (excluding dead cells). B220^+^ cells (R2 gate) within the R1 gated population was analyzed for the expression of κ light chain (M1). Non-specific staining with isotype control antibody was analyzed on live cell gate (R1’) and shown as M1’. B). Gating strategy for λ light chain expressing B cells. Live cells (R1 gate) were gated based on forward and side scatter pattern (excluding dead cells). B220^+^ cells (R2 gate) within the R1 gated population was analyzed for the expression of λ light chain (M1). Non-specific staining with isotype control antibody was analyzed on live cell gate (R1’) and shown as M1’. Quantitative data after these analyses of κ and λ light chain expression on B220^+^ cells in the bone marrow is shown in Fig 5 of the manuscript. Similar strategy gating live lymphoid population from secondary lymphoid tissues was used for analyses of κ and λ light chain on B220^+^ cells (lymph node, spleen and peyer’s patch) and IgA^+^, IgD^+^ B cells (peyer’s patch), these data shown in Fig 5 and Table 2 of the manuscript.
(TIF)
