# Supplementary figures and images for: Investigating B Cell Development, Natural and Primary Antibody Responses in Ly-6A/Sca-1 Deficient Mice
Source: PLoS One. 2016 Jun 20;11(6):e0157271. doi: 10.1371/journal.pone.0157271 (PMC4913937; doi:10.1371/journal.pone.0157271)

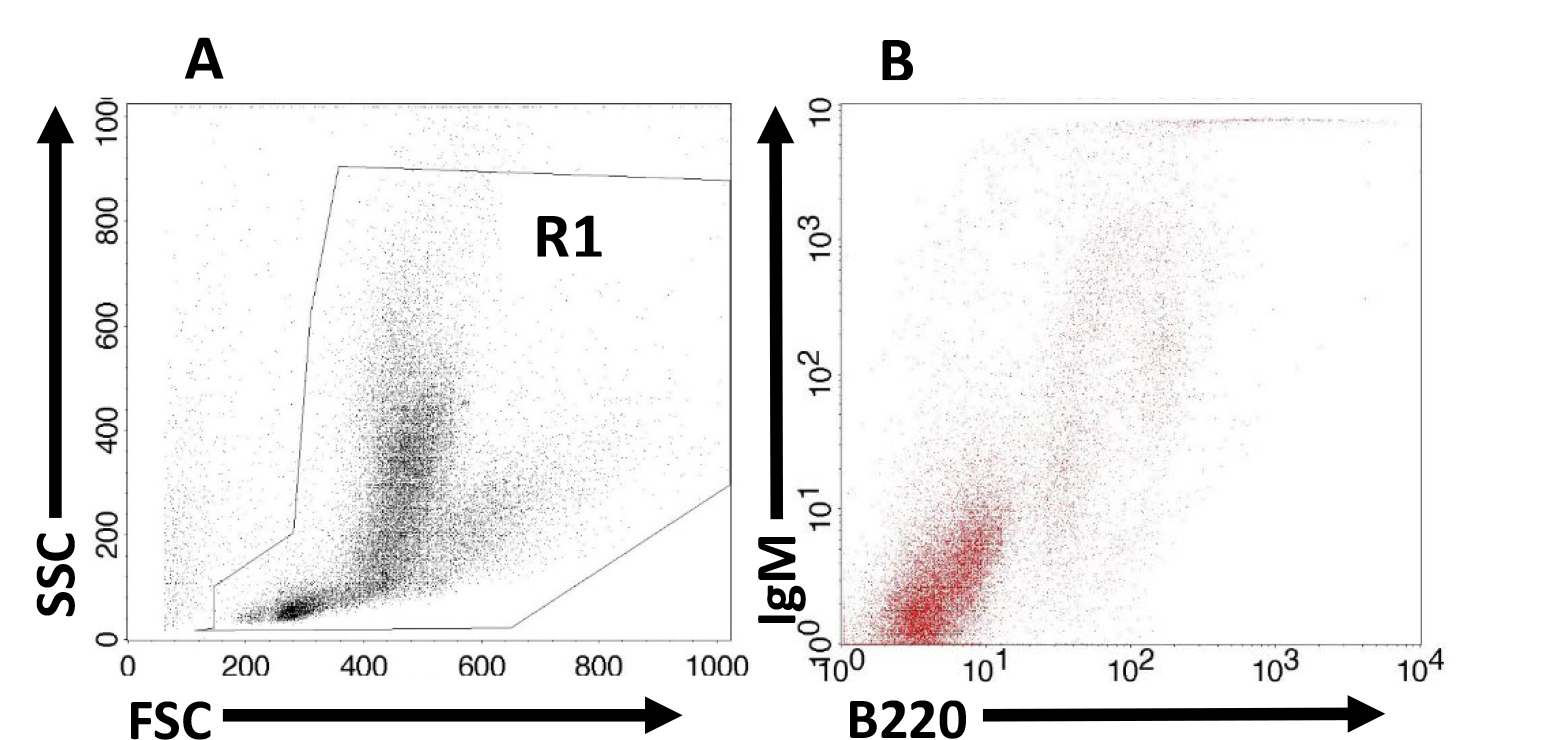

Supplement: S1 Fig — Cells were harvested from the bone marrow as described in materials and methods spleen and stained with anti-B220-FITC and anti-IgM-PE and analyzed by flow cytometer. Live lymphoid population (left panel, R1) was gated and examined for surface expression of B220 and IgM (right panel). Data with this gating strategy is shown in Figs 1 and 2. (TIF) [file pone.0157271.s001.tif]

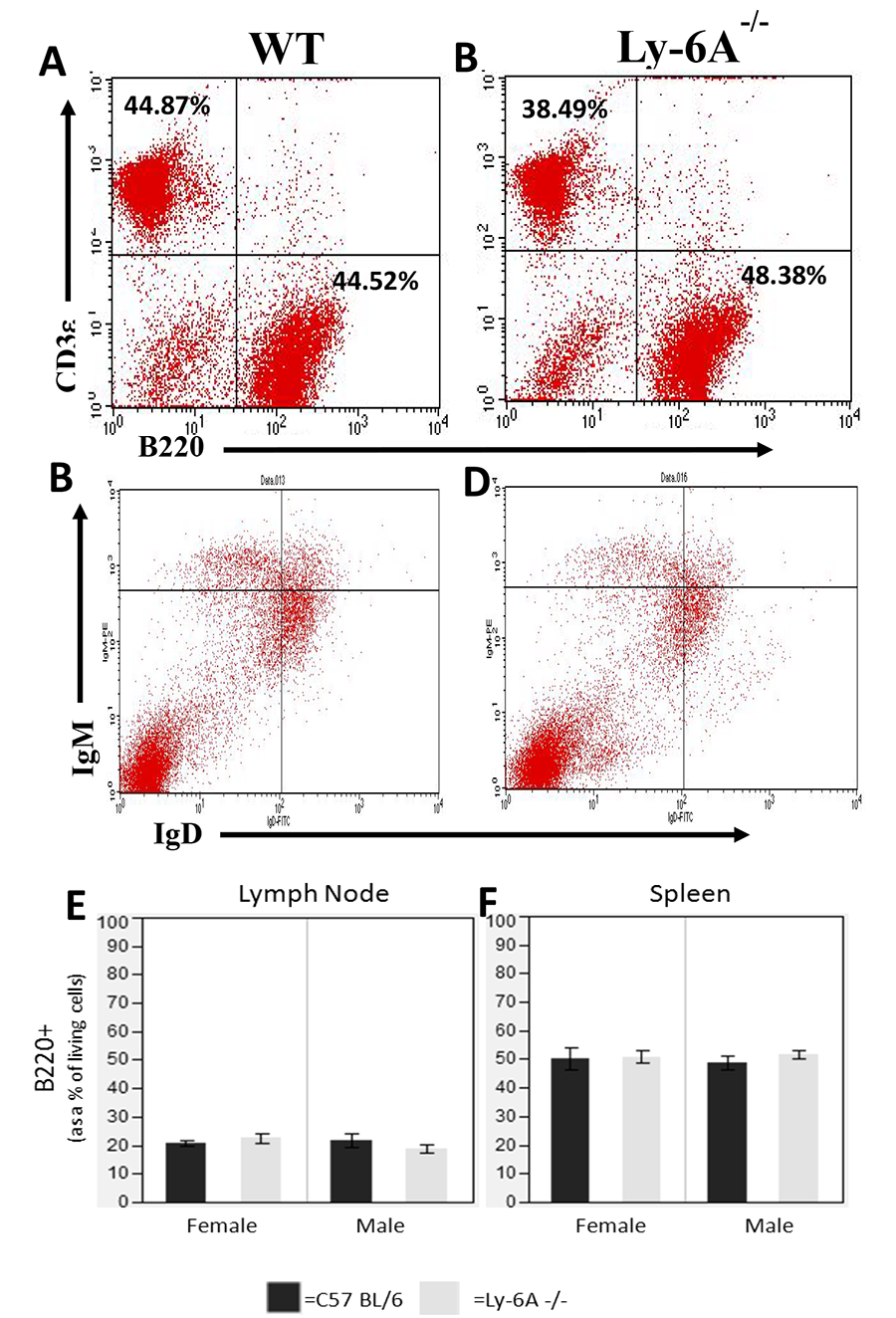

Supplement: S2 Fig — Cells were removed from the lymph nodes and spleen and stained with anti-B220 and anti-CD3ε (panels A & B) or anti-IgD and anti-IgM antibodies (panels C & D). Dot-blots for staining of live lymphoid gated spleen cells is shown (panels A-D). Cumulative data from lymph node and spleen cells is presented as a percentage of B220+ cells from gender and genotype combinations. Data represents the mean with SEM. n = 5–9 mice per genotype or sex. (TIF) [file pone.0157271.s002.tif]

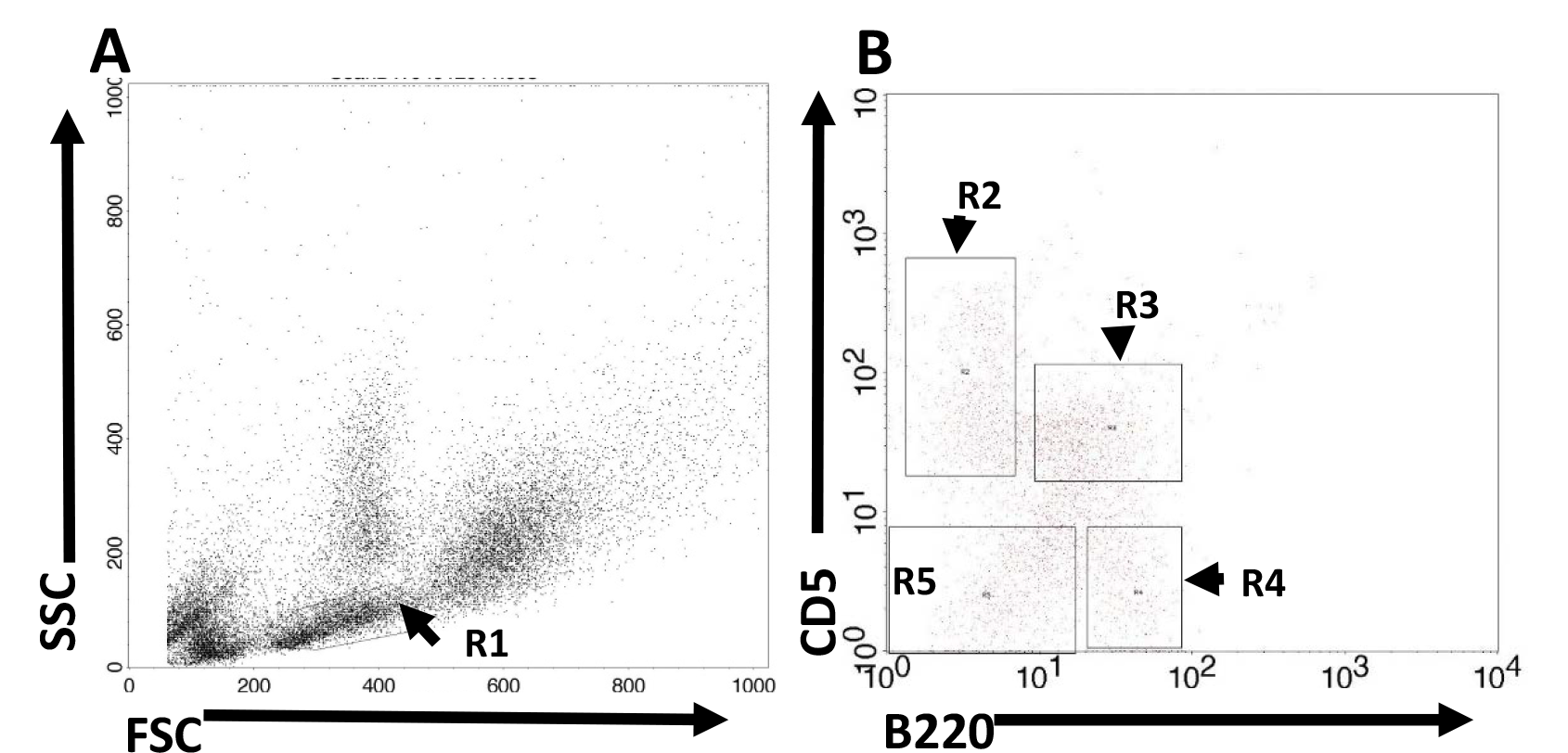

Supplement: S3 Fig — Live lymphocytes (R1) were gated based on forward and side scatter pattern (myeloid/granulocyte/ erythroid populations and dead cells were excluded) (panel A) and B-1 and B-2 B cell subsets were identified based on the expression of CD5 and B220 (panel B). B1a: CD5MedB220Low (R3); B-1b: CD5-B220Low (R5); B2: CD5-B220High (R4); T cells: CD5HighB220- (R2). Data analyses is shown in Table 1 of the manuscript. (TIF) [file pone.0157271.s003.tif]

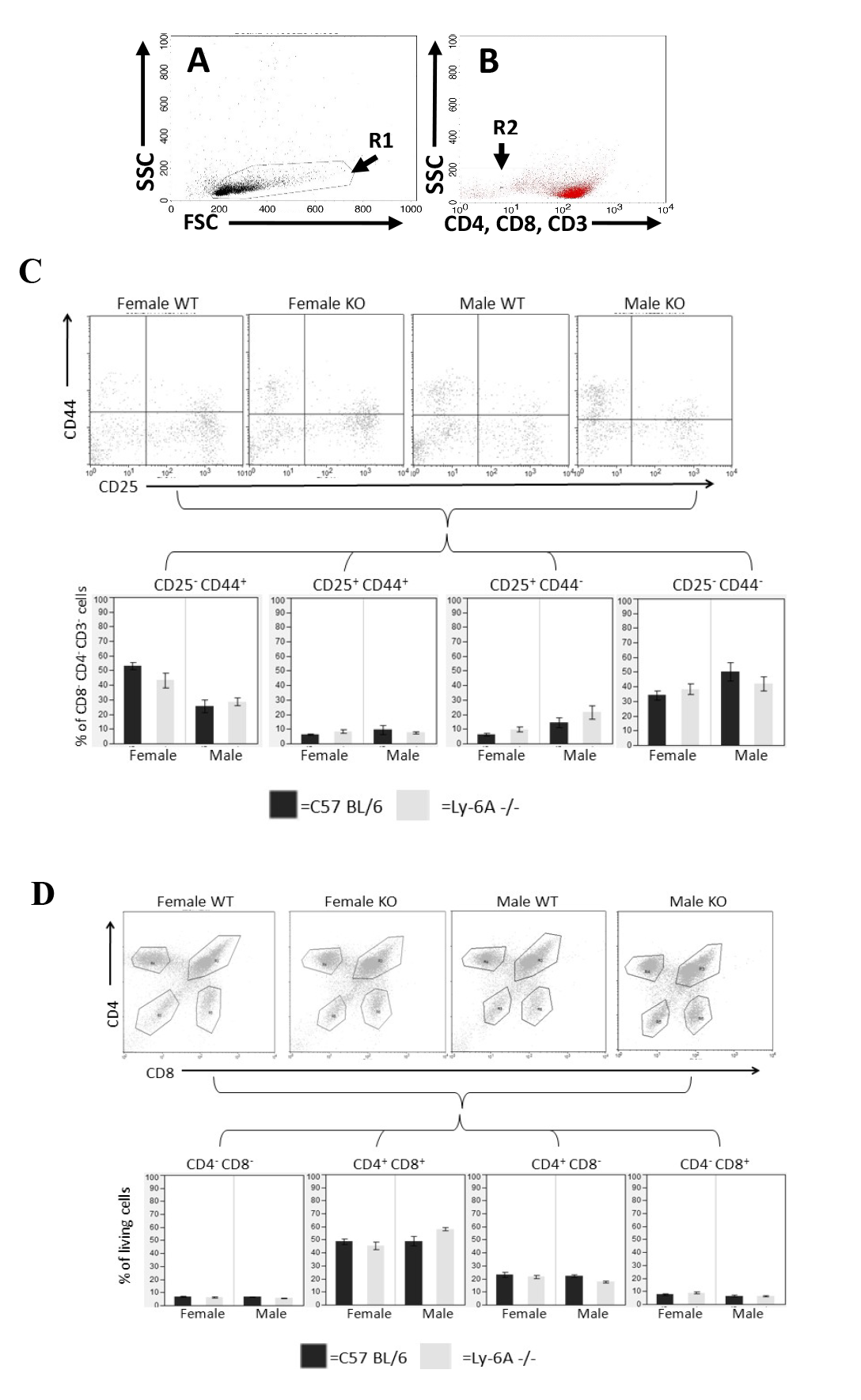

Supplement: S4 Fig — Live lymphocytes (R1) were gated based on forward and side scatter pattern (excluding dead cells) (R1 gate in panel A) and stained with anti-CD3ε, anti-CD4 and anti-CD8 (all three conjugated with same fluorophore) along with anti-CD44 and anti-CD25. The triple negative T cells (CD4-CD8- CD3-) (R2 gate in panel B) at four distinct stages of early T cell development based on the expression of CD44 and CD25 are shown (panel C). Analysis of helper and cytotoxic T cells in the thymus of Ly-6A/Sca-1 -/- mice. Percentage of living thymocytes at four distinct stages of late T cell development based on the expression of CD4 and CD8 proteins (panel D). Data is presented as a percentage of living thymocytes from sex and genotype combinations. Data represents the mean with SEM. n = 4–5 per genotype/sex. (TIF) [file pone.0157271.s004.tif]

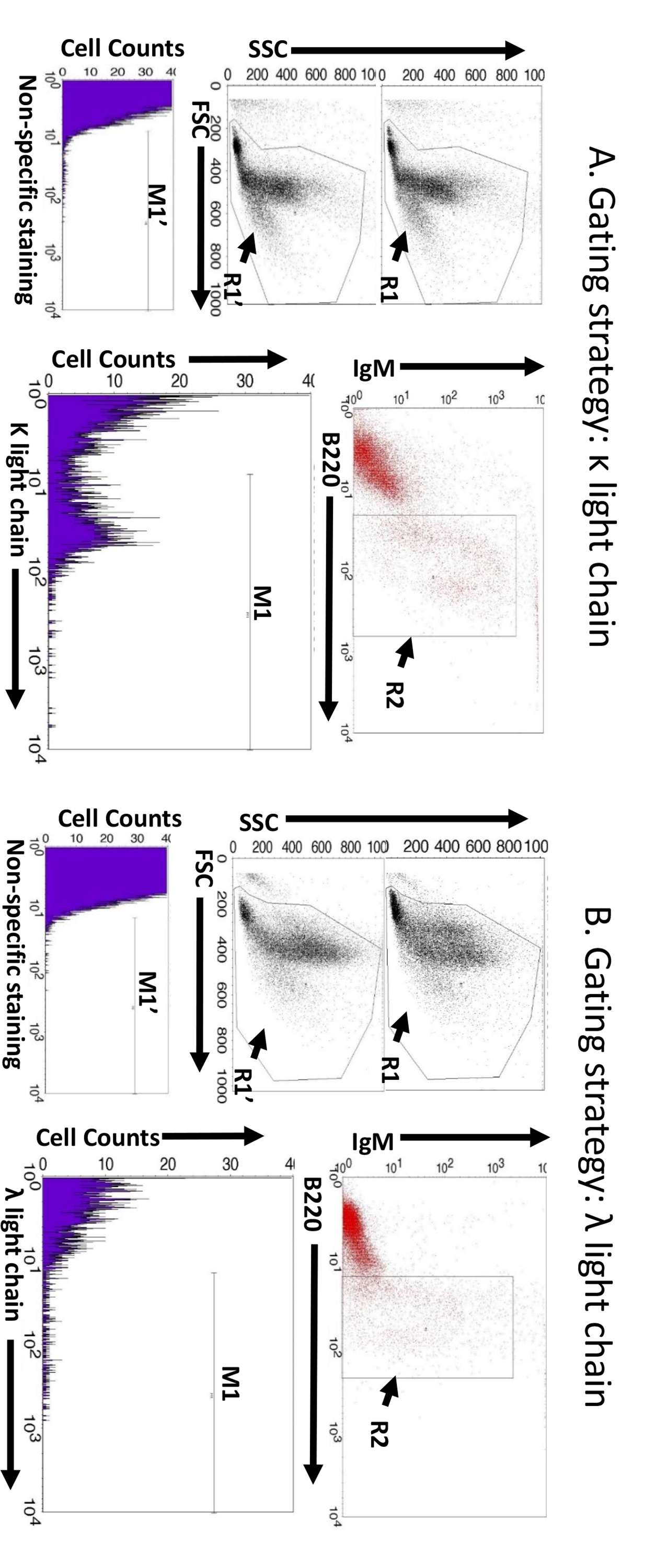

Supplement: S5 Fig — A). Gating strategy for κ light chain expressing B cells. Live cells (R1 gate) were gated based on forward and side scatter pattern (excluding dead cells). B220+ cells (R2 gate) within the R1 gated population was analyzed for the expression of κ light chain (M1). Non-specific staining with isotype control antibody was analyzed on live cell gate (R1’) and shown as M1’. B). Gating strategy for λ light chain expressing B cells. Live cells (R1 gate) were gated based on forward and side scatter pattern (excluding dead cells). B220+ cells (R2 gate) within the R1 gated population was analyzed for the expression of λ light chain (M1). Non-specific staining with isotype control antibody was analyzed on live cell gate (R1’) and shown as M1’. Quantitative data after these analyses of κ and λ light chain expression on B220+ cells in the bone marrow is shown in Fig 5 of the manuscript. Similar strategy gating live lymphoid population from secondary lymphoid tissues was used for analyses of κ and λ light chain on B220+ cells (lymph node, spleen and peyer’s patch) and IgA+, IgD+ B cells (peyer’s patch), these data shown in Fig 5 and Table 2 of the manuscript. (TIF) [file pone.0157271.s005.tif]
